# Adult Occipital Dermoid Cyst With the Initial Manifestation of Subcutaneous Lump: A Case Report

**DOI:** 10.1002/ccr3.72076

**Published:** 2026-03-19

**Authors:** Tingting Zhong, Hu Ren, Yang Xu, Mei Xiong, Xinshen Li

**Affiliations:** ^1^ Department of Dermatology The Third Hospital of Mianyang (Sichuan Mental Health Center) Mianyang Sichuan Province China; ^2^ Department of Dermatology The Affiliated Hospital of Southwest Medical University Luzhou Sichuan Province China; ^3^ Department of Science and Technology The Third Hospital of Mianyang (Sichuan Mental Health Center) Mianyang Sichuan Province China

**Keywords:** dermoid cyst, diagnosis, epidermoid cyst, surgery

## Abstract

Dermoid cysts require meticulous differential diagnosis, prompt surgical intervention, proactive management of infection and other complications, precise delineation of the lesion prior to operation, and efforts to achieve complete excision. We report a case initially diagnosed as an epidermoid cyst but finally confirmed as a dermoid cyst.

## 1 Introduction

Dermoid and epidermoid cysts have a combined incidence of about 1.6% in the head and neck [1], although both originate from the ectoderm and have squamous epithelium, only dermoid cysts contain other ectodermal structures such as hair, sebaceous glands, or sweat glands [2]. In addition, dermoid cysts are congenital and benign, being the most common cranial tumor. While epidermoid cysts are acquired and possibly malignant, being the most common cutaneous cyst [3, 4]. Dermoid cysts and epidermoid cysts often appear initially as subcutaneous nodules, which lead to confusion in diagnosis, preliminary cytological assessment of cystic lesions constitutes a pivotal component in the differential diagnostic process.

## 2 Case History and Examination

**FIGURE 1:**
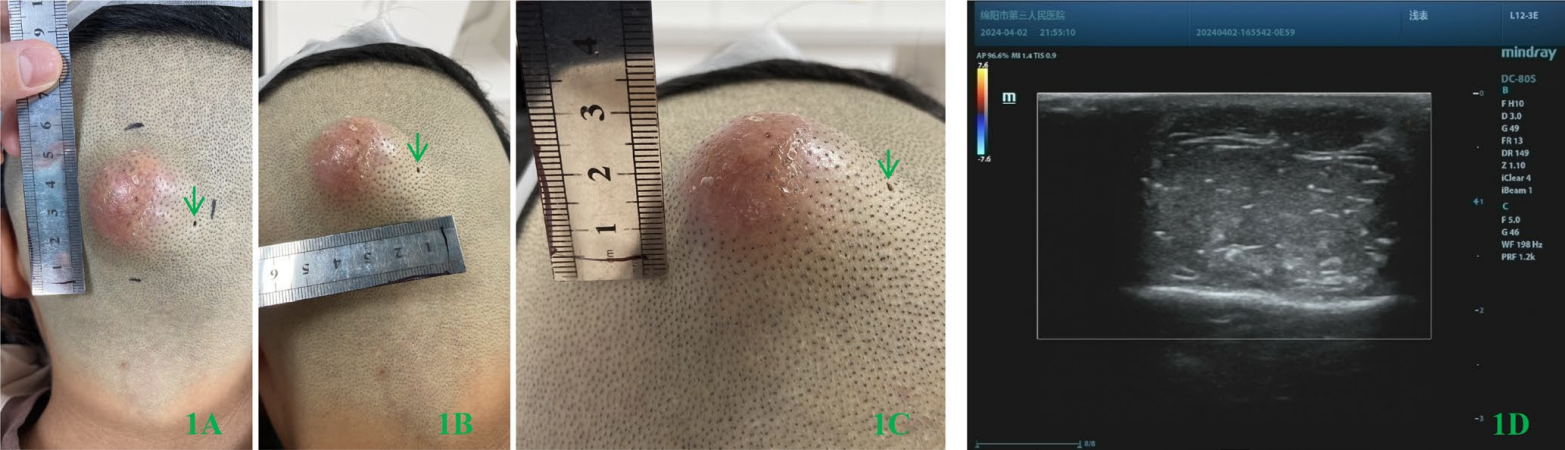
(A–C) The length, width, and height of the lump was 4.5 cm, 4.5 cm, and 3 cm, respectively. A small skin opening adjacent to the lump as indicated by the green arrow. (D) Color ultrasound indicated a hypoechoic area without blood flow signal in occipitalia scalp.

A 27‐year‐old woman presented to the dermatology department with a subcutaneous occipital lump that had been present for 20 years and had increased in size over the past 3 months. Initially, the lesion was only about 2 cm in diameter without any swelling or pain, but gradually increased to about 4 cm in diameter with mild swelling and discomfort. There were no associated symptoms such as headache, dizziness, vomiting, head trauma, or family history of similar conditions. On examination, the lesion was soft to touch with limited mobility and no significant tenderness. The boundaries were well‐defined and the skin in the area of the lump appeared relatively red and swollen. A skin opening is clearly visible adjacent to the lump (Figure 1A–C), We hypothesized that the pathogen may have induced infection via this opening. Doppler ultrasound revealed a subcutaneous occipital epidermoid cyst (Figure 1D).

## 3 Differential Diagnosis, Investigations and Treatment

**FIGURE 2:**
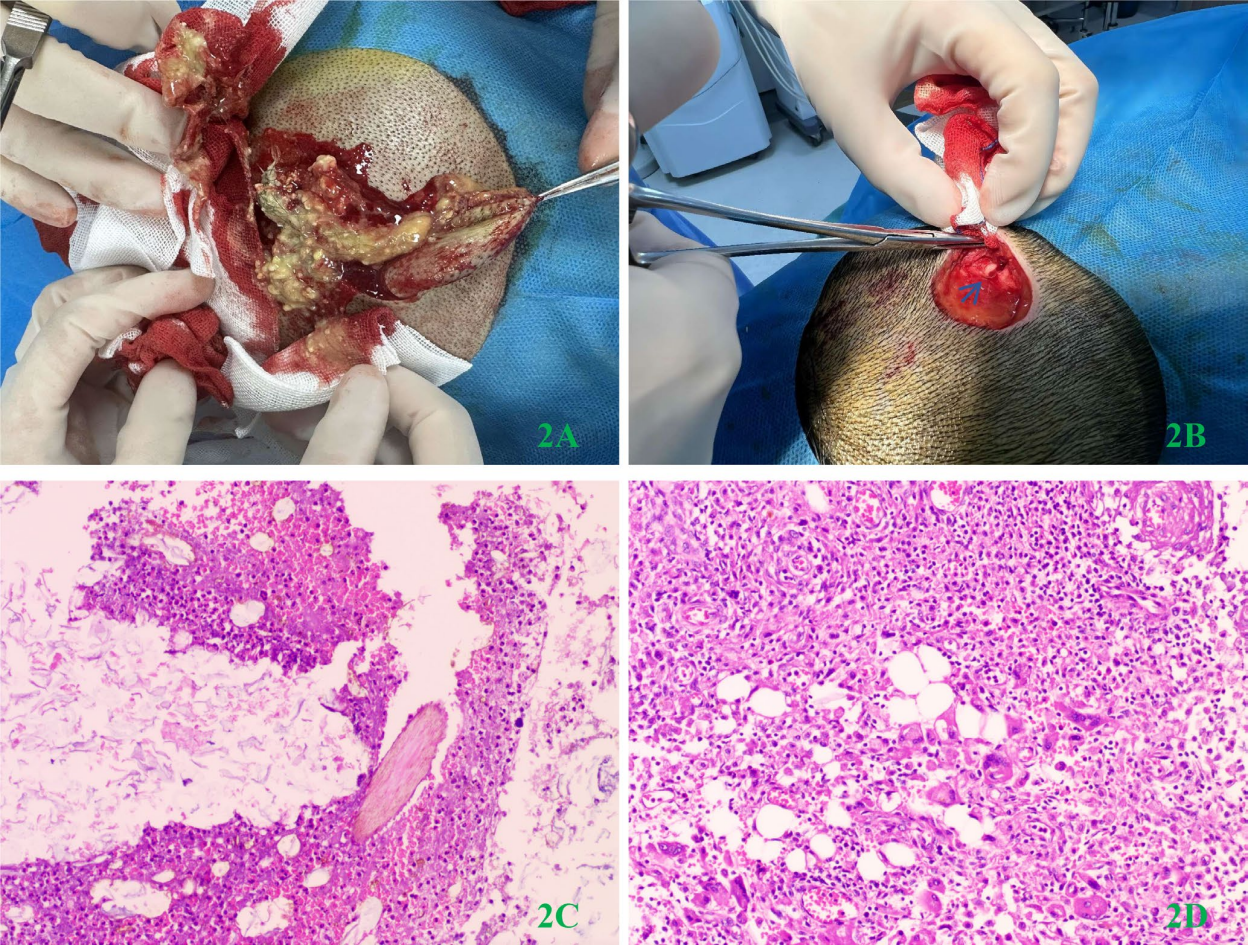
(A) Upon separation of the subcutaneous lump, a ruptured lesion accompanied by sebaceous glands and hair flow was observed without a visible envelope. (B) Upon clearing the effluent, a skull defect measuring 0.5 cm*1 cm was identified, within the residue could not be cleared, as indicated by the blue arrow. (C) The effluent contained inflammatory necrotic tissue, keratinocytes, and hair (Ratio of 1:100). (D) Infiltration of plasma cells and multinuclear giant cells in the dermis with the formation of granuloma was observed (Ratio of 1:100).

The patient was initially diagnosed with a subcutaneous occipital epidermoid cyst, with her informed consent, we performed the excision of the occipital scalp lesion under local anesthesia in the minor operation room. During the procedure, no visible envelope was observed when separating the lump, which resulted in rupture of the mass along with sebaceous glands and hair flow. When the effluent was cleared, a skull defect was discovered, and the residue could not be removed. The surgical process and pathological examination results are depicted in Figure 2A,B and Figure 2C,D, respectively.

**FIGURE 3:**
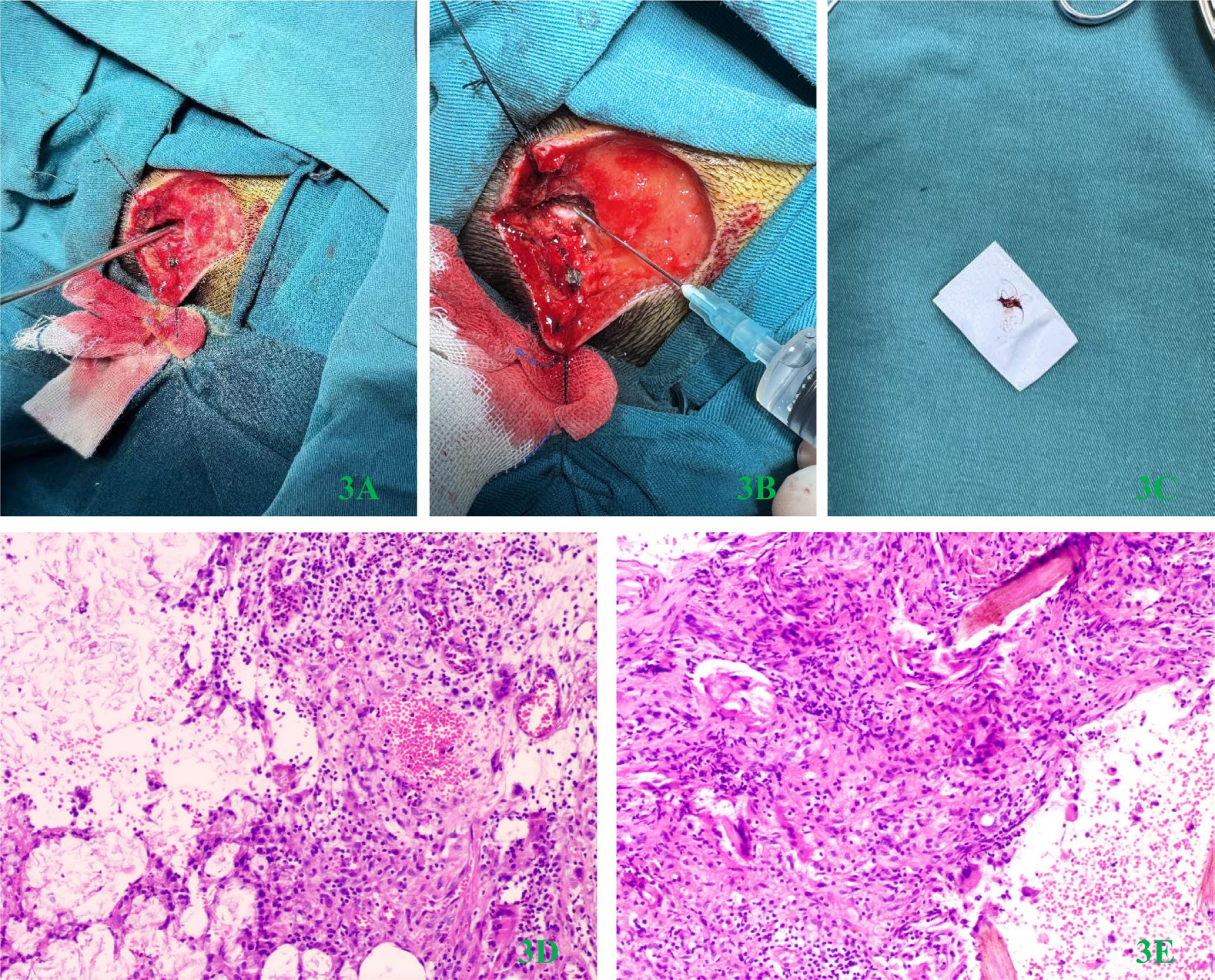
(A) The lesion penetrated the outer plate of the occipital bone, resulting in a defect. (B) The defect was subsequently enlarged and cleared of sebaceous glands and hair components. (C) Hair components were extracted from the skull defect area. (D) Inflammatory cells and keratinocytes were observed within the lesion (Ratio of 1:100). (E) Hair and multinucleated giant cells infiltrated the affected region (Ratio of 1:100).

We then revised the diagnosis to occipital dermoid cyst, and the neurosurgeon performed a dissection of the occipital lesion. Intraoperative exploration revealed that the lesion had invaded the outer plate of the occipital bone but had not penetrated the skull. After enlarging the skull defect to the size of 1 cm*2 cm by careful grinding, circular bone destruction containing sebaceous glands and hair was observed in the outer plate of the occipital bone. The lesion was cleared and examined pathologically, and the wound was washed with 3% hydrogen peroxide, 5% Povidone iodine solution, and normal saline alternately. The minimal cortical defect in the skull was intentionally preserved to facilitate the observation of potential lesion recurrence. The second surgical process and pathological examination results are shown in Figure 3A–C and Figure 3D,E, respectively.

## 4 Outcome and Follow‐Up

**FIGURE 4:**
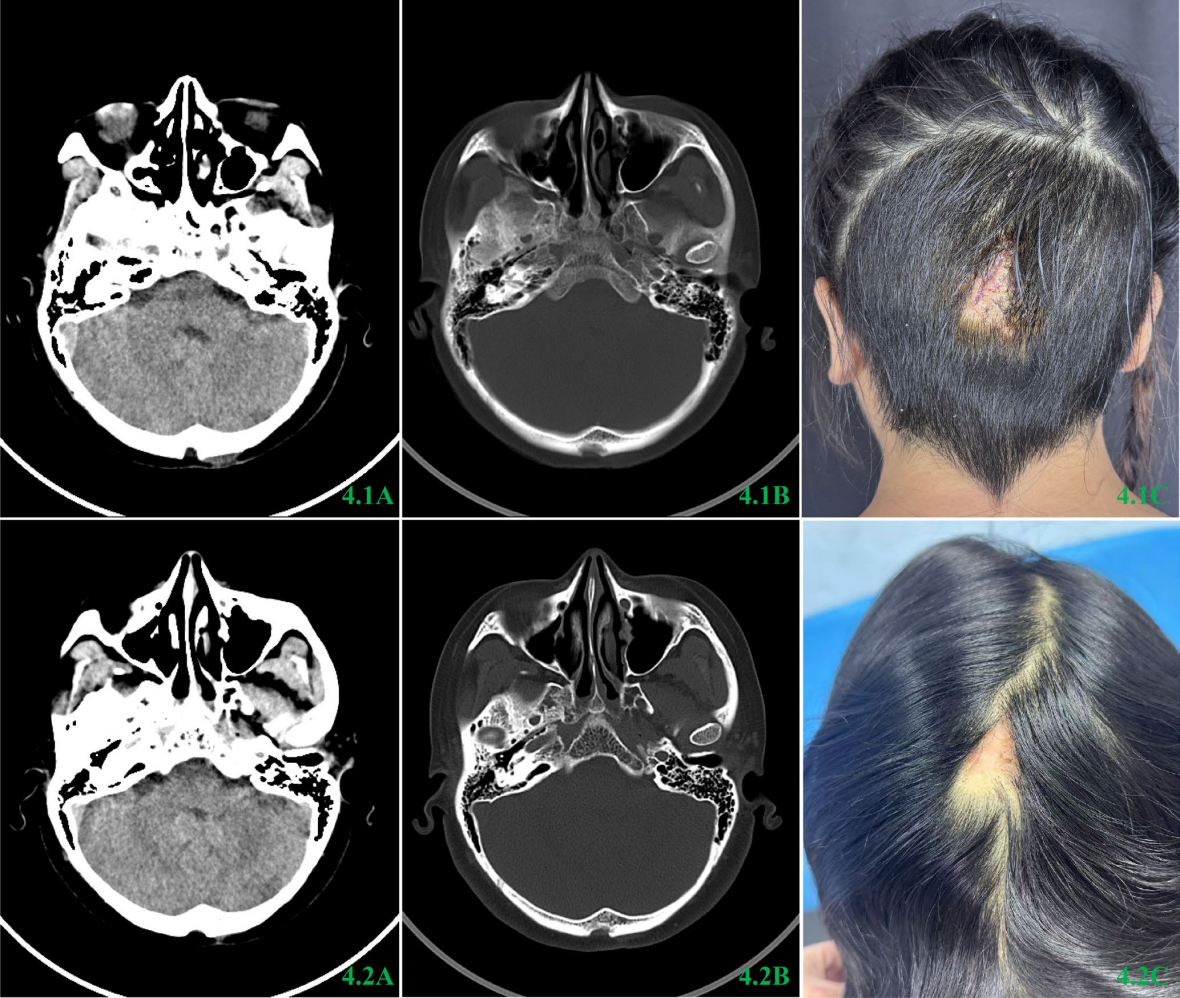
4.1A–C and 4.2A–C refer to 14 days and 10 months after the secondary wound closure, respectively. In 4.1A and 4.2A, CT showed that subcutaneous occipital and occipital lesions had been cleared; no definite abnormality was found in the brain parenchyma. In 4.1B and 4.2B, a short strip defect was observed near the outer plate of the occipital bone. In 4.1C and 4.2C, the incision was anastomosed and healed well.

Given the high probability that the dermoid cyst was complicated by infection, suturing was done after 5 days without the symptom of infection. After a 14‐day and 10‐month period of secondary wound closure, craniocerebral CT scan revealed complete resolution of subcutaneous occipital and occipital lesions, with a partial defect in the outer plate of the occipital bone. The patient's incision had fully healed, and she opted not to undergo treatment for the cortical defect of the skull. The CT examination results and the appearance of the incision after suture removal are depicted in Figure 4.

## 5 Discussion

Dermoid cysts are rare developmental malformations that arise from abnormal wrapping of surface ectoderm along the skin fusion line during embryonic development; they are typically found along skull suture lines or at anterior fontanels on the head and face [5]. In this case, the dermoid cyst was located in the middle suture of the occipital bone, which is consistent with its embryonic development.

Occipital dermoid cysts are even rarer than other types and can be difficult to discern from epidermoid cysts. and needs to be differentiated from teratomas, sebaceous cysts, lipomas, scalp hemangiomas and Turban tumor syndrome [4, 6]. Unlike epidermoid cysts, dermoid cysts contain ectodermal appendage structures such as sweat glands, sebaceous glands, hair follicles, and hair. Teratomas are characterized by the presence of abnormally differentiated tissues originating from all three germ layers, typically containing lipid‐like substances, hair, teeth, and osseous structures. MRI serves as a valuable diagnostic tool for the identification. Sebaceous gland cysts can be presented as hemispherical uplift, and the contents are mainly sebaceous. Lipomas appear as flat lobulated subcutaneous adipose tissue masses that have a soft texture and good mobility. Scalp hemangioma refers to racemose distributions of scalp vascular malformation, which can be identified by Doppler ultrasound [7, 8, 9, 10, 11]. Dermoid cysts exhibit characteristics of slow progression, deep midline distribution, wide base, limited mobility, absence of tenderness, and a palpable cystic neoplasm. In this case, the rapid growth of the occipital dermoid cyst over the past 3 months may be attributed to changes in sex hormone levels within the body, particularly an increase in androgen levels that can stimulate sebaceous gland secretion and epithelial cell formation [12].

The extent of dermoid cyst involvement requires special attention, as some cases with prolonged disease duration may involve cranial or even intracranial regions leading to intracranial‐extracranial communicating dermoid cyst formation. Surgical intervention is the recommended treatment for dermoid cysts, and preoperative CT imaging aids in devising a comprehensive surgical plan for complete excision. Failure to completely remove the dermoid cyst and leaving behind any remnants of its wall increases the risk of recurrence post‐surgery. Early diagnosis and timely surgical intervention, especially during childhood, are crucial to prevent secondary infections associated with dermoid cysts while also reducing treatment duration and improving prognosis [12].

## Author Contributions

**Tingting Zhong:** conceptualization, methodology, project administration, visualization, writing – original draft. **Hu Ren:** data curation, resources. **Yang Xu:** supervision, visualization. **Mei Xiong:** data curation, investigation. **Xinshen Li:** formal analysis, investigation, software, validation, writing – review and editing.

## Funding

This study is supported by the research project of The Third Hospital of Mianyang (No. 202240); Sichuan Medical Association Taige research project (No. 2021TG54).

## Ethics Statement

Written informed consent was obtained from the patient for publishing the case report, and the research was approved by the Ethics Committee of The Third Hospital of Mianyang on May 27, 2024.

## Conflicts of Interest

The authors declare no conflicts of interest.

## Data Availability

All datasets on which the conclusions of the paper rely are available to editors, reviewers and readers without unnecessary restriction. The data that support the findings of this study are not publicly available due to the privacy of research participants, but are available from the corresponding author XL upon reasonable request.

## References

[1] J. Pupić‐Bakrač , A. Pupić‐Bakrač , I. Bačić , M. Š. Kolega , and N. Skitarelić , “Epidermoid and Dermoid Cysts of the Head and Neck,” Journal of Craniofacial Surgery 32 (2021): e25–e27. 32796308 10.1097/SCS.0000000000006834

[2] D. Zhao , Y. Han , Y. Chen , and J. Qiu , “An Unusual Dermoid Cyst in Subcutaneous Tissue of the Mastoid Region: A Case Report,” Experimental and Therapeutic Medicine 6 (2013): 75–76. 23935722 10.3892/etm.2013.1080 PMC3735589

[3] J. J. Taylor , A. G. Scherer , L. Shao , and T. J. Westmoreland , “Concurrent Dermoid and Epidermoid Cysts in an Adolescent Patient: A Case Report,” Oxford Medical Case Reports 2023 (2023): omad105. 37881261 10.1093/omcr/omad105 PMC10597612

[4] A. Prior , P. Anania , M. Pacetti , et al., “Dermoid and Epidermoid Cysts of Scalp: Case Series of 234 Consecutive Patients,” World Neurosurgery 120 (2018): 119–124. 30189303 10.1016/j.wneu.2018.08.197

[5] D. Reissis , M. J. Pfaff , A. Patel , and D. M. Steinbacher , “Craniofacial Dermoid Cysts: Histological Analysis and Inter‐Site Comparison,” Yale Journal of Biology and Medicine 87 (2014): 349–357. 25191150 PMC4144289

[6] J. Bargiel , G. Wyszyńska‐Pawelec , M. Gontarz , et al., “Turban Tumor Syndrome: In Search of a Gold Standard ‐ A Case Report,” Skin Appendage Disorders 7 (2021): 326–328. 34307484 10.1159/000514855 PMC8280440

[7] J. Woodley‐Cook , M. MacDonald , J. Karamchandani , J. Spears , and A. Bharatha , “Epidermoid Cyst of the Central Nervous System With Marked Lipid Content,” Clinical Neuroradiology 25 (2015): 317–320. 10.1007/s00062-014-0344-0 25287159

[8] H. M. Yoon , S. J. Byeon , J. Y. Hwang , et al., “Sacrococcygeal Teratomas in Newborns: A Comprehensive Review for the Radiologists,” Acta Radiologica 59 (2018): 236–246. 28530139 10.1177/0284185117710680

[9] M. Ludovici , N. Kozul , S. Materazzi , R. Risoluti , M. Picardo , and E. Camera , “Influence of the Sebaceous Gland Density on the Stratum Corneum Lipidome,” Scientific Reports 8 (2018): 11500. 30065281 10.1038/s41598-018-29742-7 PMC6068117

[10] Y. Katsuyama , T. Shirai , R. Terauchi , et al., “Chondroid Lipoma of the Neck: A Case Report,” BMC Research Notes 11 (2018): 415. 29954455 10.1186/s13104-018-3523-2 PMC6022339

[11] T. H. Kim , J. W. Choi , and W. S. Jeong , “Current Concepts of Vascular Anomalies,” Archives of Craniofacial Surgery 24 (2023): 145–158. 37654234 10.7181/acfs.2023.00332 PMC10475703

[12] J. Bajric , G. J. Griepentrog , and B. G. Mohney , “Pediatric Periocular Dermoid Cysts: Incidence, Clinical Characteristics, and Surgical Outcomes,” Ophthalmic Epidemiology 26 (2019): 117–120. 30260262 10.1080/09286586.2018.1525412 PMC6839760

